# Mechanical Behaviour of Aluminium-Timber Composite Connections with Screws and Toothed Plates

**DOI:** 10.3390/ma15010068

**Published:** 2021-12-22

**Authors:** Marcin Chybiński, Łukasz Polus

**Affiliations:** Institute of Building Engineering, Faculty of Civil and Transport Engineering, Poznan University of Technology, Piotrowo 5 Street, 60-965 Poznan, Poland

**Keywords:** aluminium-timber structures, laminated veneer lumber (LVL), toothed plate, screwed connection, shear connection, push-out test

## Abstract

This paper presents an investigation of the load-slip behaviour of aluminium-timber composite connections. Toothed plates with bolts are often used for connecting timber structural members with steel structural members. In this paper, toothed plates (C2-50/M10G, C2-50/M12G or C11-50/M12) have been used as reinforcement in aluminium-timber screwed connections for the first time. The push-out test specimens consisted of laminated veneer lumber slabs, aluminium alloy beams, and hexagon head wood screws (10 mm × 80 mm and 12 mm × 80 mm). Of the specimens, 12 additionally had toothed plates as reinforcement, while 8 had no reinforcement. The load carrying-capacity, the mode of failure and the load-slip response of the strengthened and non-strengthened screwed connections were investigated. The use of toothed plate connectors was found to be effective in increasing the strength of aluminium-timber composite connections and ineffective in improving their stiffness. The examined stiffness and strength of the connections can be used in the design and numerical modelling of aluminium-timber composite beams with reinforced screwed connections.

## 1. Introduction

Currently, great importance is attached to civil engineering solutions being sustainable. The use of timber and engineered wood products in the construction industry reduces the carbon footprint. Growing trees absorb CO_2_ from the atmosphere. Furthermore, wood products require less fossil fuels to be produced than other building materials, such as steel [1]. The limitations of sawn timber were overcome after the development of engineered wood products, such as glued-laminated timber, cross-laminated timber and laminated veneer lumber [2]. Recent scientific studies on timber structures can be divided into four groups: material tests (e.g., [3,4]), connections for timber elements (e.g., [5,6]), strengthening of timber elements (e.g., [7,8,9]), and composite structures with timber structural members.

A composite beam consists of two or more structural elements which are permanently joined [10]. Timber can be combined with non-wood building materials, e.g., with steel [11,12], concrete [13,14], aluminium [15,16] or glass [17,18]. Furthermore, structural elements made of wood-based materials can also be combined with each other [19]. Recently, the experimental behaviour of timber-concrete and steel-timber composite structures has been investigated in a number of studies [20,21,22]. However, the behaviour of aluminium-timber composite structures has only been studied in a few tests [23,24,25,26,27]. The behaviour of composite elements depends on their connections. In a simply supported composite beam, the slab is designed to resist compression, the girder is designed to resist tension, while shear is transferred through connectors referred to as “shear connectors”. There are many types of shear connections used in composite beams with timber elements (see Table 1). 

Screws with hexagonal heads may be used in laterally loaded connections [31]. A large diameter of a hexagonal head wood screw maximises screw resistance against head pull-through. Self-tapping screws are optimised for loading in the axial direction and they can be installed without pre-drilling [32]. The installation of self-tapping screws in cold-formed steel beams is relatively simple. However, when composite beam girders are made of beams with thick flanges, the installation of self-tapping screws requires the use of additional elements to connect the slabs with the girders [33].

The results of experimental tests on aluminium-timber composite beams with screwed connections were presented in [25]. Hexagon head wood screws were used to join an aluminium beam with an LVL slab. The failure mode of the analysed screwed connections was associated with the crushing of the timber, the formation of one plastic hinge within the connector, and the hole ovalisation in the aluminium beam flange. The stiffness and strength of the connection per one connector were relatively low (*k*_0.4_ = 5.5 kN/mm, *P_ult_* = 15.1 kN). For this reason, the authors of this paper proposed to use a toothed plate in the screwed connection. The main goal of this paper was to determine the stiffness and the load-carrying capacity of the screwed connection with the toothed plate.

## 2. Materials and Methods

### 2.1. LVL

The material parameters of LVL are presented in Table 2. The engineered wood product was fabricated from Scots pine (*Pinus sylvestris* L.) and Norway spruce (*Picea abies* L. H. Karst) veneers [34].

### 2.2. Aluminium Alloy

The mechanical properties of the AW-6060 T6 aluminium alloy were determined in a tensile test [36] and are presented in [37] (see Table 3).

### 2.3. Shear Connectors

**Figure 1:**
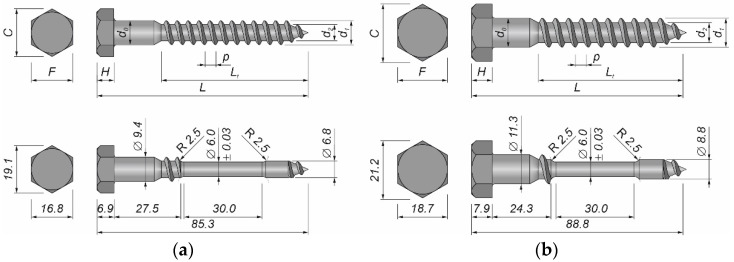
A shear connector: (**a**) 10 mm screw; (**b**) 12 mm screw.

Grade 5.8 hot dip galvanised DIN 571 [38] hexagon head wood screws 10 mm × 80 mm and 12 mm × 80 mm were used as shear connectors (see Table 4 and Table 5). The mechanical properties of the steel used in the screws were determined experimentally in accordance with [36]. Four round samples were created from 10 mm screws and another four round samples were created from 12 mm screws for the purpose of the tensile tests. The thread was removed in the middle of the screw to obtain a smooth shank and to install the extensometer on the sample (see Figure 1).

The tensile tests were conducted using an Instron 4483 machine (Instron, HighWycombe, Buckinghamshire, UK) and an Epsilon 3442-010M-025M-ST extensometer (Epsilon, Jackson, WY, USA) with a 10 mm gauge. The displacement rate was kept constant (0.05 mm/s).

### 2.4. Toothed Plates

Toothed-plate connectors (C2-50/M10G, C2-50/M12G or C11-50/M12) were used to reinforce the aluminium-timber screwed connections investigated in this paper.

**Figure 2:**
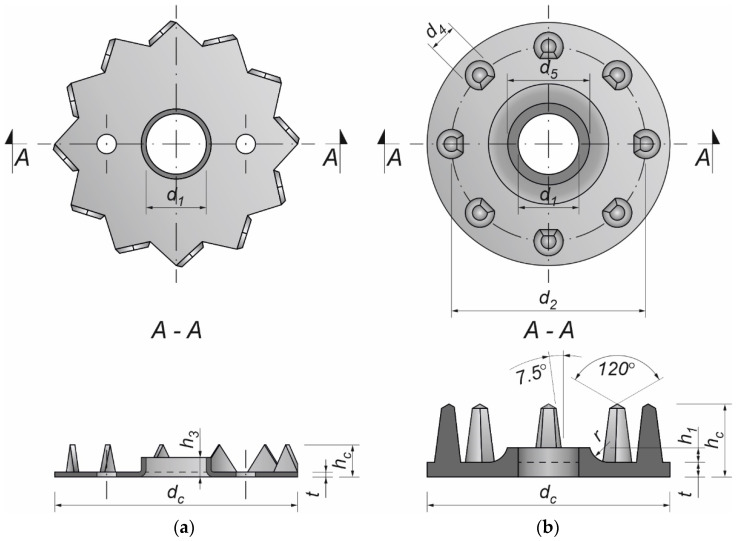
Toothed-plate connectors: (**a**) type C2, Bulldog; (**b**) C11, Geka.

A toothed plate (type C2, Bulldog) is a single-sided connector made from a circular plate. Its edges are cut and bent over to form triangular teeth projecting from one face at 90° to the face (see Figure 2a). Around the screw hole, there is a flange projecting from the same face as the teeth. The dimensions of the toothed plates used in this study are presented in Table 6. They were made from cold rolled uncoated low carbon narrow strips of HC340LA steel (high yield strength steel for cold forming) [39]. The toothed plates were hot dip galvanised (≥45 μm) to protect them from corrosion.

A toothed-plate connector (type C11-50/M12, Geka) is a single-sided connector made from a round plate with spikes on one side of the plate (see Figure 2b). The spikes are equidistant and are arranged in one circle. The toothed plate has a bolt-hole through its centre with a flange around the bolt-hole projecting from the same face as the spikes. The dimensions of the toothed plates used in this study are presented in Table 7. They were made of malleable cast iron EN-GJMB-350-10 (PN-JM 1130) according to EN 1562 [40] and galvanised (Fe/Zn12/C) to protect them from corrosion.

### 2.5. Push-Out Tests

**Figure 3:**
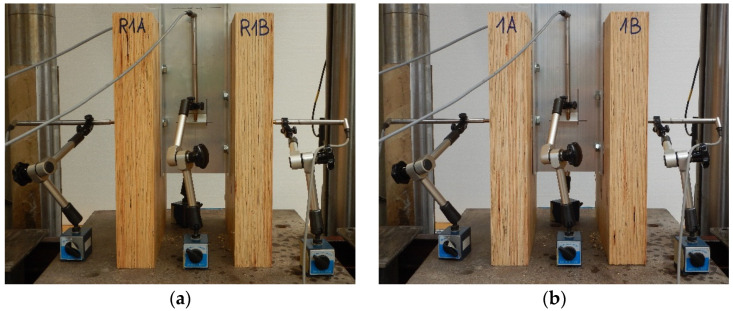
The tested specimens: (**a**) without reinforcing toothed plates; (**b**) with reinforcing toothed plates.

The tests were carried out on twenty models using an Instron 8505 Plus machine (In-stron, High Wycombe, Buckinghamshire, UK). Each experimental model consisted of two timber slabs made of LVL and a beam made of the AW-6060 T6 aluminium alloy (see Figure 3). The LVL slabs were connected with the aluminium beams using four variants of connections. In the first variant, eight hexagon head wood screws (10 × 80 mm^2^) without reinforcing toothed plates were used (specimen R10.1–R10.4). In the second variant, eight hexagon head wood screws (10 × 80 mm^2^) with reinforcing toothed plates (C2-50/M10G, Bulldog) were used (specimens 10.1–10.4). In the third variant, eight hexagon head wood screws (12 × 80 mm^2^) without reinforcing toothed plates were used (specimen R12.1–R12.4). In the four variant, eight hexagon head wood screws (12 × 80 mm^2^) with reinforcing toothed plates (C2-50/M12G, Bulldog) were used (specimens 12.1–12.4). In the fifth variant, eight hexagon head wood screws (12 × 80 mm^2^) with reinforcing toothed plates (C11-50/M12, Geka) were used (specimens 12.5–12.8).

**Figure 4:**
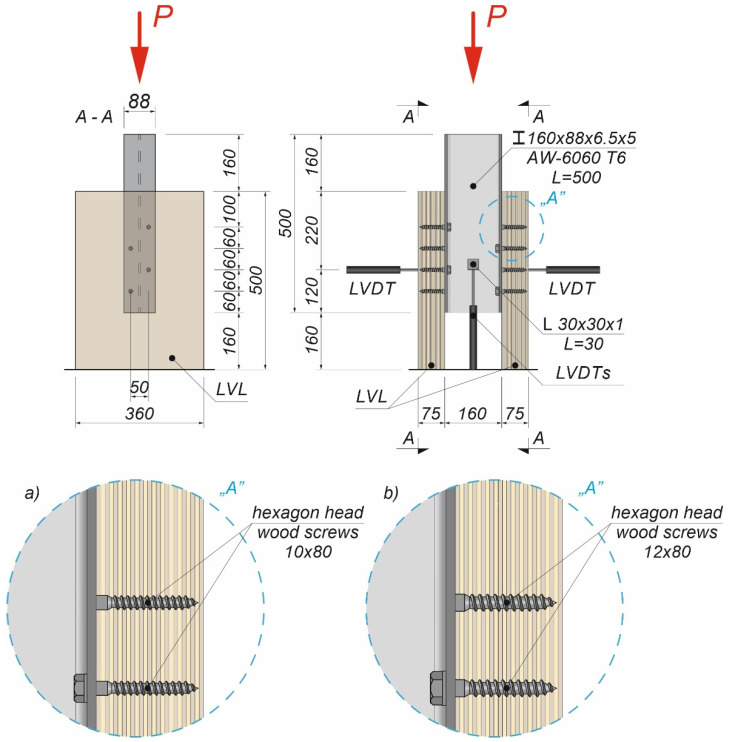
The location of the LVDTs on the specimen without reinforcing toothed plates: (**a**) screws 10 × 80; (**b**) screws 12 × 80.

**Figure 5:**
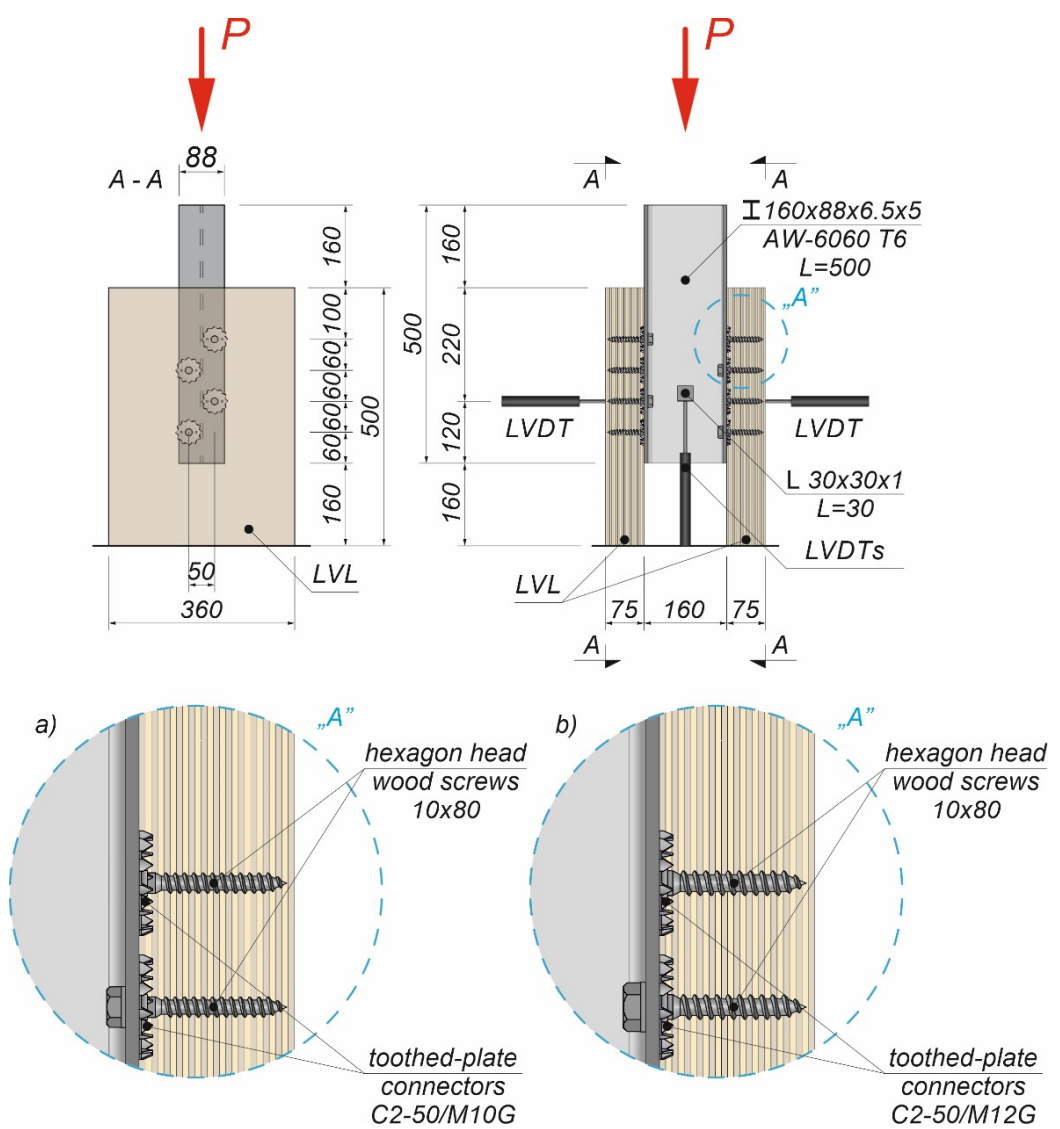
The location of the LVDTs on the specimen with reinforcing toothed plates: (**a**) screws 10 × 80 and toothed-plate connectors (type C2-50/M10G); (**b**) screws 12 × 80 and toothed-plate connectors (type C2-50/M12G, Bulldog).

**Figure 6:**
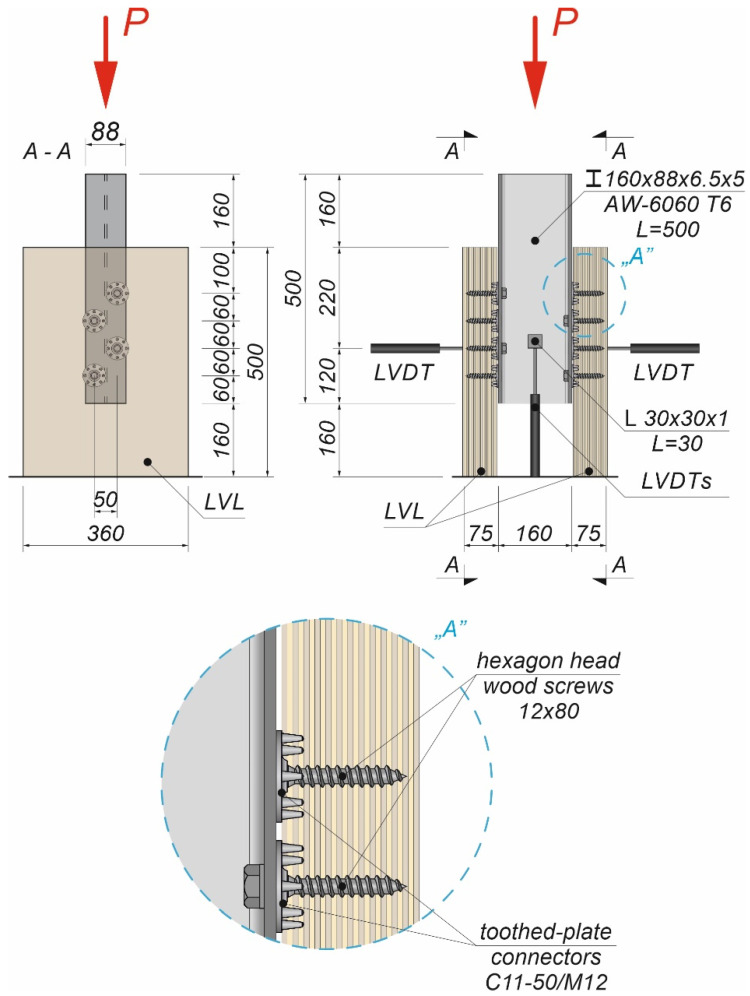
The location of the LVDTs on the specimen with reinforcing toothed plates—screws 12 × 80 and toothed-plate connectors (type C11-50/M12, Geka).

The holes in the aluminium beams had the same diameter as the screws to reduce the slip between the aluminium beams and the LVL slabs. The pre-drilling diameter in LVL was 7 mm for the 10 mm screw and 8 mm for the 12 mm screw. Pre-drilling started the course of the screw and created pilot holes. Furthermore, the installation of the hexagon head wood screws required less effort. In each specimen, screws were inserted in the face withdrawal direction using a torque wrench (Sandvik Belzer, IZO-I-100, 10–100 Nm). The tread-grain angle was 90°. The torque level was measured during the insertion of the screws using a torque wrench and recorded at the end of the insertion process (35 Nm for 10 mm screw, 50 Nm for 12 mm screw). The toothed plates were pressed into LVL using a hydraulic press and a compressive force equal to 35 kN. The spaces between the screws were 50 mm in the transverse direction and 60 mm in the longitudinal direction. The staggered spacing was used because of the dimensions of the toothed plates. The loading direction was parallel to the LVL grain. Linear variable differential transformers (LVDTs) were used to measure the longitudinal slip between the LVL slabs and the aluminium beam, and the horizontal move of the sample (see Figure 4, Figure 5 and Figure 6).

The push-out tests were performed in line with [44]. In the first part of the test, a load control regime was applied to achieve a regular shape of the load–slip curve and to determine the connection slip modulus for a shear force equal to 40% of *F_max_*. In the second part of the test, a constant rate of displacement was used to evaluate the behaviour of the connection once the ultimate load had been achieved. The shear force was first increased from 0 to 40% of *F_est_* over two minutes, and it remained at this level for the next 30 seconds. Afterwards the load was reduced from 40% to 10% of *F_est_* and kept at this level for additional 30 seconds. Subsequently, the load was increased from 10% to 70% of *F_est_*. Up to that point, the push-out tests were performed using a load control regime, and from then on—using a displacement control regime (the piston velocity was 5.0 mm/min). The ultimate load *F_est_* = 130.0 kN was calculated based on Equation (8.10e) from Eurocode 5 [45]. The value of *F_est_* was modified during the tests taking into account the previous results. The loading procedure was also redefined.

## 3. Results and Discussion

### 3.1. The Results of the Tensile Tests of the Steel Used in the Screws

The tensile strength of the steel used in the screws was 553.9 ± 23.6 MPa (4.3%) [46]. The measurement error for the tensile strength of the steel used in the screws was determined using Student’s t-distribution with 7 degrees of freedom and a confidence level of 95%.

### 3.2. The Results of the Push-Out Tests

**Figure 7:**
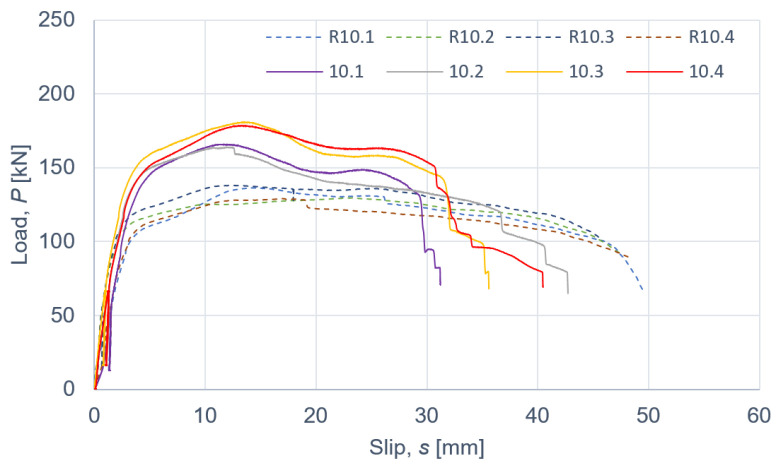
The load–slip curves from the push-out tests of the shear connections with 10 mm screws and with toothed-plate connectors (type C2-50/M10G, Bulldog) in specimens 10.1–10.4 or without toothed-plate connectors in specimens R10.1-R10.4.

**Figure 8:**
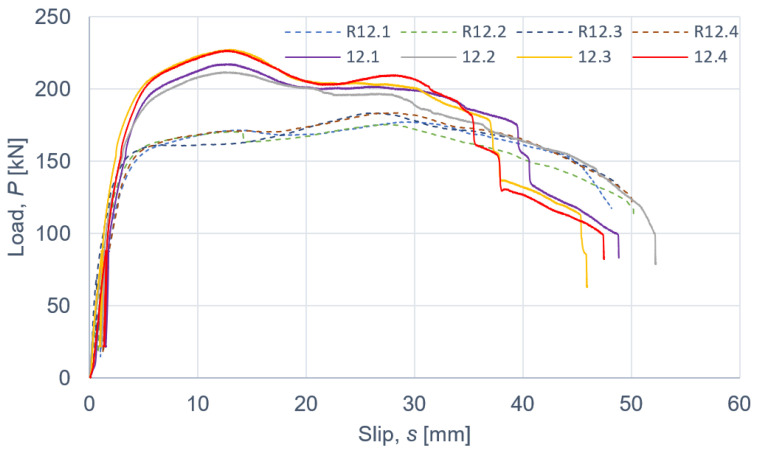
The load–slip curves from the push-out tests of the shear connections with 12 mm screws and with toothed-plate connectors (type C2-50/M12G, Bulldog) in specimens 12.1–12.4 or without toothed-plate connectors in specimens R12.1-R12.4.

**Figure 9:**
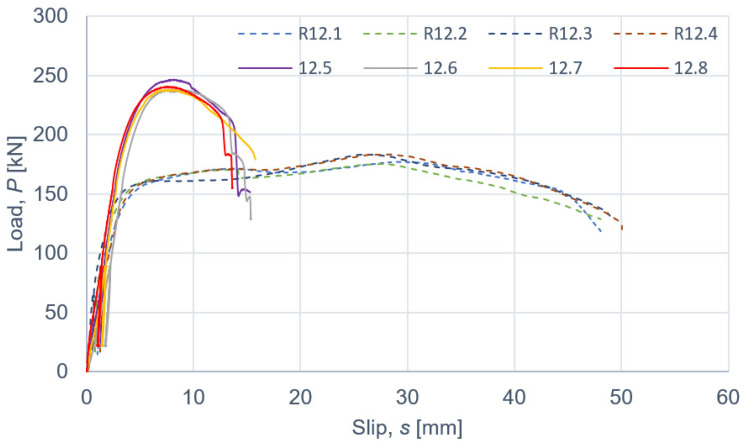
The load–slip curves from the push-out tests of the shear connections with 12 mm screws and with toothed-plate connectors (type C11-50/M12, Geka) in specimens 12.5–12.8 or without toothed-plate connectors in specimens R12.1-R12.4.

The results of the push-out tests are presented in Figure 7, Figure 8 and Figure 9 and in Table 8, Table 9, Table 10, Table 11 and Table 12. The symbols used in Table 8, Table 9, Table 10, Table 11 and Table 12 are as follows: *P_ult_*, ultimate load per one connector; *s_ult_*, slip corresponding to *P_ult_*; *k*_0.4_ and *k*_0.6_, slip moduli per one connector. The measurement errors presented in Table 8, Table 9, Table 10, Table 11 and Table 12 were determined using Student’s t-distribution with 3 degrees of freedom and a confidence level of 95%.

Taking into account the results of the specimens without the reinforcing toothed plates and comparing them with the mean ultimate load and the mean slip modulus of the specimens with the reinforcing toothed plates, the below conclusions were drawn. 

The use of toothed-plate connectors in aluminium-timber composite connections can enhance their load-carrying capacity. An enhancement of 28.7% (for 10 mm screws and C2-50/M10G toothed-plate connectors), 23.8% (for 12 mm screws and C2-50/M12G toothed-plate connectors) or 35.0% (for 12 mm screws and C11-50/M12 toothed-plate connectors) was achieved in the respective screwed connections. Upon comparing the slip moduli of the tested connections, it was observed that the use of toothed plate connectors was ineffective in improving the stiffness of the aluminium-timber composite connections. 

According to Eurocode 4 [47], a connection is ductile if its characteristic slip capacity is at least 6 mm. All the tested connections had the characteristic slip capacity exceeding 6 mm. However, the screwed connections with the C11-50/M12 toothed-plate connectors (Geka) had a brittle mode of failure—the unthreaded part of the screw was sheared. The screwed connections with the C2-50/M12G toothed-plate connectors (Bulldog) were more ductile than the screwed connections with the C11-50/M12 toothed-plate connectors (Geka) (compare Figure 8 and Figure 9). There was a single shear plane between the toothed-plate connectors and the aluminium beam flange. The stiffness of the Geka toothed-plate connector is higher than the stiffness of the Bulldog toothed-plate connector because the flange height of the former (6 mm) is 1.5 times higher than the flange height of the latter (4 mm), and the flange thickness of the former (2.25 mm) is 2.25 times higher than the thickness of the latter (1 mm). Furthermore, the thickness of the former (3 mm) is 3 times higher than the thickness of the latter (1 mm). In the case of the screwed connections with the C11-50/M12 toothed-plate connectors (Geka), the screws were sheared, whereas in the case of the screwed connections with the C2-50/M12G toothed-plate connectors (Bulldog), the toothed-plate connectors were torn.

The load-carrying capacity of the screwed connections with the C2-50/M12G toothed-plate connectors (Bulldog) (27.6 kN) was 1.09 times lower than the load-carrying capacity of the screwed connections with the C11-50/M12 toothed-plate connectors (Geka) (30.1 kN).

**Figure 10:**
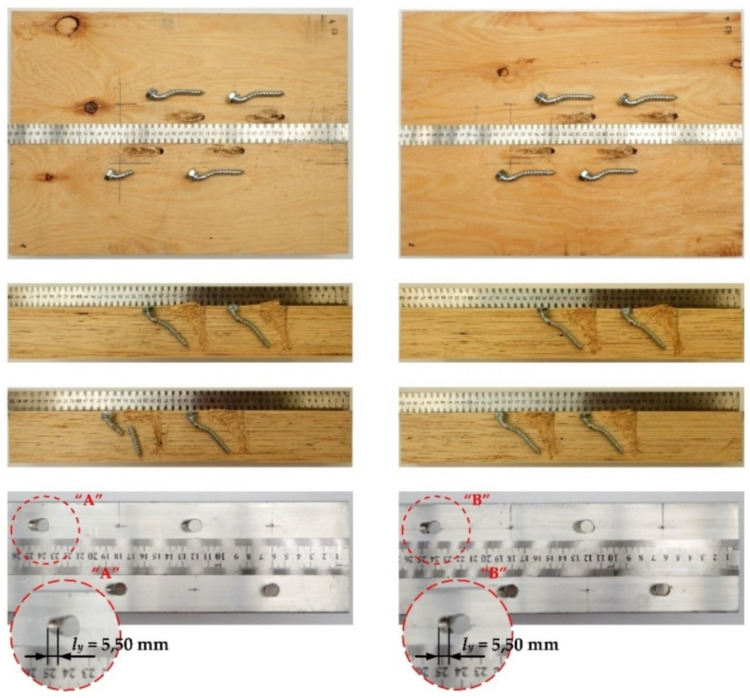
The mode of failure of the aluminium-timber connection with the 10 mm screws and without the reinforcing toothed plates.

**Figure 11:**
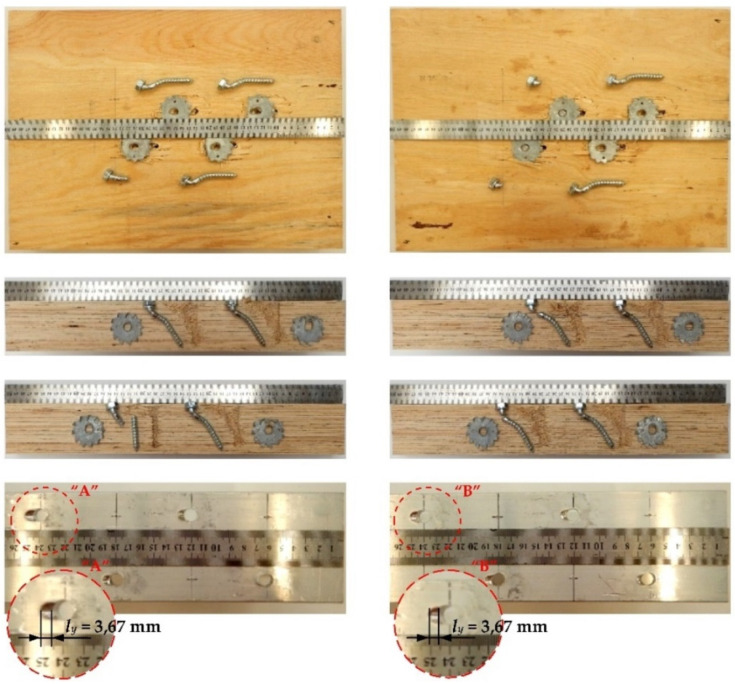
The mode of failure of the aluminium-timber connection with the 10 mm screws and the reinforcing toothed plates (C2-50/M10G, Bulldog).

**Figure 12:**
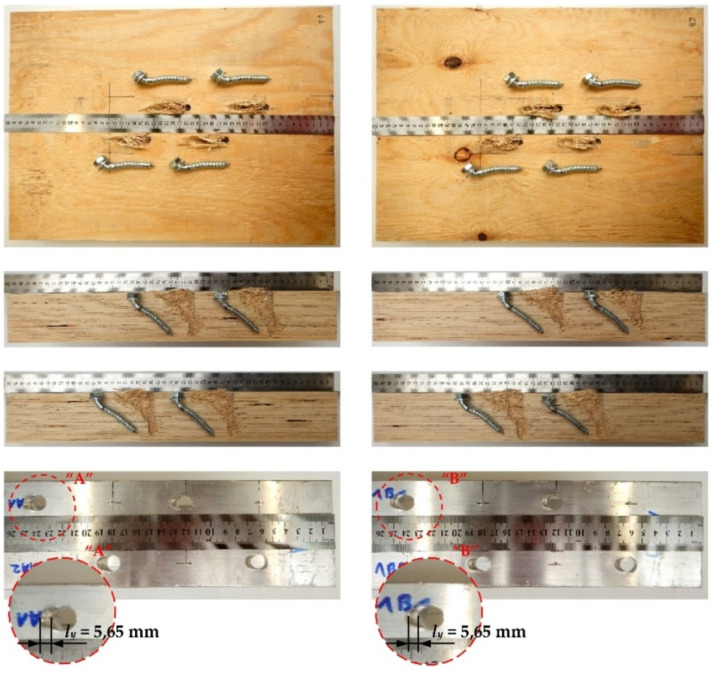
The mode of failure of the aluminium-timber connection with the 12 mm screws and without the reinforcing toothed plates.

**Figure 13:**
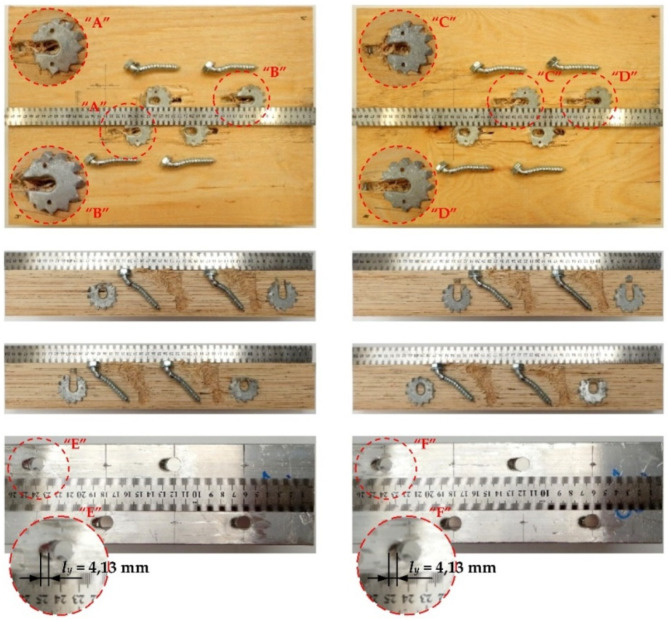
The mode of failure of the aluminium-timber connection with the 12 mm screws and the reinforcing toothed plates (C2-50/M12G, Bulldog).

**Figure 14:**
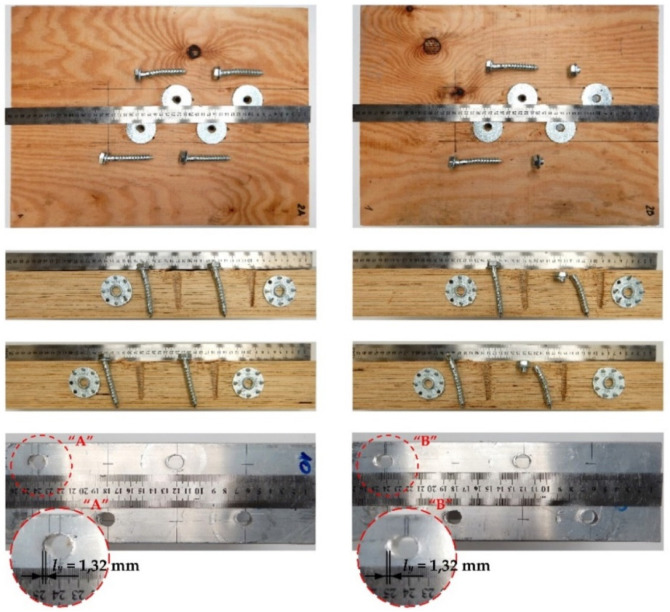
The mode of failure of the aluminium-timber connection with the 12 mm screws and the reinforcing toothed plates (C11-50/M12, Geka).

The tested screwed connections with or without Bulldog toothed-plate connectors (C2-50/M10G, C2-50/M12G) showed one distinctive mode of failure presented in Figure 10, Figure 11, Figure 12 and Figure 13. The authors observed the formation of two plastic hinges within the screw, the crushing of LVL, hole ovalisation in the flange of the aluminium alloy beam, and hole ovalisation in the toothed plate or its tearing. In the specimens where the teeth were strongly connected with the LVL and did not allow for the movement of the toothed plates, the toothed plates were torn (see Figure 11). Some of the screws were sheared near the end of the tests. In Figure 10, Figure 11, Figure 12, Figure 13 and Figure 14, the symbol *l_y_* was used to present the mean length of the yielded zone in the aluminium flange (measured at the end of the tests). The yielded zone was caused by the bearing of the screw to the hole wall.

The tested screwed connections with Geka toothed-plate connectors (C11-50/M12) showed one distinctive mode of failure presented in Figure 14. The screws were sheared and some of the plate teeth were broken. The authors also observed the crushing of LVL and the hole ovalisation in the flange of the aluminium alloy beam. In the case of Geka toothed-plate connectors, the mean length of the yielded zone in the aluminium flange was shorter than in the Bulldog toothed-plate connectors. The connections with the Geka toothed-plates had a lower slip corresponding to the ultimate load than the connections with the Bulldog toothed-plates.

The failure mode of the tested screwed connections with or without Bulldog toothed-plate connectors is taken into account in Equation (8.10e) presented in [45]:

(1) $$P_{v,Rk}=2.3\sqrt{M_{y,Rk}f_{h,k}d}+\frac{F_{ax,Rk}}{4}$$

where: *P_v_*_,*Rk*_ is the characteristic load-carrying capacity of the screw in a single shear (13.3 kN for the 9.43 mm screw and 17.5 kN for the 11.31 mm screw) calculated from Equation (1), *f_h_*_,*k*_ is the characteristic embedment strength of the timber (40.8 MPa for the 9.43 mm screw and 40.0 MPa for the 11.31 mm screw) calculated from [45], *t* is the penetration depth (70.0 mm in this paper), *M_y_*_,*Rk*_ is the characteristic fastener yield moment (54 372 N·mm for the 9.43 mm screw and 87 226 N·mm for the 11.31 mm screw) calculated from [45], *f_u_*_,*k*_ is the characteristic tensile strength of the screw (530.3 MPa—5%-quantile from the tensile tests), and *F_ax_*_,*Rk*_ is the characteristic withdrawal capacity of the fastener (11,011 N for the 9.43 mm screw and 12 059 N for the 11.31 mm screw) calculated from [45].

The mean values of the screw shank diameters (9.43 mm for the 10 mm screw and 11.31 mm for the 12 mm screw) from Table 3 and Table 4 were used in the calculations based on Equations (1)–(4).

The characteristic load-carrying capacity of the 9.43 mm screw calculated from Equation (1) (13.3 kN) was 1.29 times lower than the ultimate load per one screw in the screwed connection without the reinforcing toothed plate (17.1 kN) and 1.62 times lower than the ultimate load per one screw in the reinforced screwed connection (21.5 kN). The characteristic load-carrying capacity of the 11.31 mm screw calculated from Equation (1) (17.5 kN) was 1.22 times lower than the ultimate load per one screw in the screwed connection without the reinforcing toothed plate (21.4 kN) and 1.58 times lower than the ultimate load per one screw in the reinforced screwed connection (27.6 kN). The model presented in Eurocode 5 does not take into account the reinforcing toothed plate. For this reason, the values obtained from Equation (1) are similar to the values obtained in the tests of the screwed connections without the reinforcing toothed plates, and lower than the ones from the tests with the reinforcing toothed plates.

The failure mode of the tested screwed connections with Geka toothed-plate connectors (C11-50/M12), i.e., the shearing of the unthreaded part of the screw, is taken into account in the equation presented in Table 3.4 in [48]:

(2) $$P_{v,Rk}=\frac{\alpha_{v}f_{ub}A}{\gamma_{M2}}$$

where: *P_v_*_,*Rk*_ is the characteristic load-carrying capacity of the screw in a single shear (26.7 kN for the 11.31 mm screw) calculated from Equation (2), α_v_ is the coefficient from [48] (0.6), *A* is the gross cross-section area of the connector, *γ_M_*_2_ is the partial safety factor, *f_ub_* is the ultimate strength of the steel used in the shear connector.

The characteristic load-carrying capacity of the 11.31 mm screw calculated from Equation (2) (26.7 kN) was 1.03 times lower than the ultimate load per one screw in the screwed connection without the reinforcing toothed plate (27.6 kN) and 1.13 times lower than the ultimate load per one screw in the reinforced screwed connection (30.1 kN). The model presented in Eurocode 5 does not take into account the reinforcing toothed plate. For this reason, the values obtained from Equation (2) are similar to the values obtained in the tests of the screwed connections without the reinforcing toothed plates, and lower than the values from the tests with the reinforcing toothed plates.

Hassanieh et al. [49] presented the formulae that can characterise the load-carrying capacity of the steel-timber screwed connection (per one connector).

(3) $$P_{ult}=(5.95d-27.2)/2$$

where: *P_ult_* is the ultimate load per one connector (14.5 kN for the 9.43 mm screw and 20.0 kN for the 11.31 mm screw) calculated from Equation (2), *d* is the screw diameter.

Steel-timber composite structures are similar to aluminium-timber composite ones. The characteristic load-carrying capacity of the 9.43 mm screw calculated from Equation (3) (14.5 kN) was 1.18 times lower than the ultimate load per one screw in the screwed connection without the reinforcing toothed plate (17.1 kN) and 1.48 times lower than the ultimate load per one screw in the reinforced screwed connection (21.5 kN). The characteristic load-carrying capacity of the 11.31 mm screw calculated from Equation (3) (20.0 kN) was 1.07 times higher than the ultimate load per one screw in the screwed connection without the reinforcing toothed plate (21.4 kN) and 1.38 times lower than the ultimate load per one screw in the reinforced screwed connection (27.6 kN). The model presented by Hassanieh et al. [49] does not take into account the reinforcing toothed plate, neither does the model presented in Eurocode 5. The results of the tests presented in this article show that reinforcing LVL by toothed-plate connectors is effective in increasing the load-carrying capacity of screwed connections. An enhancement of 23.8%, 28.7%, or 35.0% was achieved in the screwed connections with 10 or 12 mm screws, respectively. For this reason, the authors of this paper suggested Equation (3) be modified by adding a coefficient of 1.24, taking into account the lowest value of the enhancements obtained from the tests, to characterise the load-carrying capacity of the aluminium-timber screwed connection reinforced with toothed-plate connectors:

(4) $$P_{ult}=1.24(5.95d-27.2)/2$$

The characteristic load-carrying capacity of the 9.43 mm screw calculated from Equation (4) (18.0 kN) was 1.19 times lower than the ultimate load per one screw in the reinforced screwed connection (21.5 kN). The characteristic load-carrying capacity of the 11.31 mm screw calculated from Equation (4) (24.9 kN) was 1.11 times lower than the ultimate load per one screw in the screwed connection reinforced by the C2-50/M12G toothed plates (27.6 kN) and 1.21 times lower than the ultimate load per one screw in the screwed connection reinforced by the C11-50/M12 toothed plates (30.1 kN). 

In this paper, toothed plates were used as reinforcement. However, LVL can also be reinforced using other steel elements. For example, Hassanieh et al. [49] used reinforcing nail plates. They compared the load-carrying capacity and the stiffness of steel-timber screwed connections with and without nail plates. Hassanieh et al. [49] showed that the use of nail plates increased the stiffness of the connection, e.g., by 22% for 16 mm screws. What is more, reinforcing the LVL slab by nail plates enhanced the load-carrying capacity of the connection, e.g., by 19% for 16 mm screws. They also observed that nail plates had a minor influence on the load-carrying capacity of the steel-timber composite connections loaded in the direction perpendicular to the grain. The influence of the toothed plates (C2-50/M10G, C2-50/M12G, C11-50/M12) used in the tests presented in this article on the load-carrying capacity of the aluminium-timber screwed connections was similar to the impact of the nail plates used by Hassanieh et al. [49] on the load-carrying capacity of the steel-timber screwed connections.

## 4. Conclusions

In this paper, the load-carrying capacity, stiffness, load-slip response, failure modes and ductility of aluminium-timber screwed connections with and without toothed plates were investigated. Push-out tests with symmetrical configurations were conducted. 

Based on the results of the tests, the following conclusions can be drawn. Aluminium-timber screwed connections can be reinforced using toothed plates. Reinforcing LVL by toothed-plate connectors can enhance the load-carrying capacity of screwed connections. Enhancements of 28.7% (for 10 mm screws and C2-50/M10G toothed-plate connectors), 23.8% (for 12 mm screws and C2-50/M12G toothed-plate connectors) or 35.0% (for 12 mm screws and C11-50/M12 toothed-plate connectors) were achieved in the screwed connections. However, the use of toothed plate connectors was found to be ineffective in improving the stiffness of aluminium-timber composite connections.

The authors demonstrated that the existing design rules did not take into account the strengthening effect of toothed plates on the connection load-carrying capacity, and they suggested the use of a coefficient equal to 1.24 to better characterise the load-carrying capacity of aluminium-timber screwed connections reinforced with toothed-plate connectors.

Furthermore, the screwed connections reinforced with toothed plates may be used in aluminium-timber composite beams. The tests presented in this paper make it possible to determine the number of connectors necessary to achieve the required level of composite action. Last, but not least, the obtained load-slip curves for the analysed connections can be used in numerical models of aluminium-timber composite beams, to model connection behaviour using spring elements. This method of connection modelling was used, e.g., in [25,27,30,50,51].

## Figures and Tables

**Table 1:** Shear connections used in composite beams with timber elements.

Composite Beam	Shear Connection	Example
steel-timber	self-drilling screw [11]	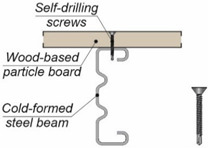
steel-timber	coach screw [21]	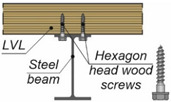
steel-timber	coach screw [21]	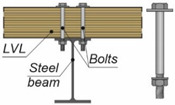
aluminium-LVL	hexagon head wood screw [25]	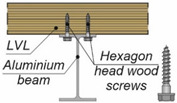
aluminium-LVL	bolt [27]	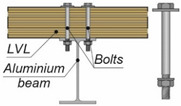
LVL-concrete	rectangular notch reinforced with a coach screw [28]	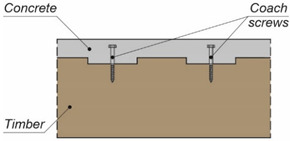
timber-timber	coach screw [29]	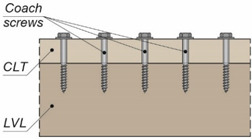
timber-timber	fully threaded inclined screw [30]	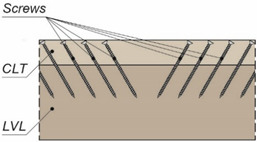

**Table 2:** The material parameters of LVL [35].

Material Parameters	Value
Mean value of modulus of elasticity (parallel to grain) *E*_0,*mean*_ [MPa]	14,000
Bending strength (flatwise, parallel to grain) *f_m_*_,0,*flat,k*_ [MPa]	50.0
Tension strength (parallel to grain) *f_t_*_,0,*k*_ [MPa]	36.0
Compression strength (parallel to grain) *f_c_*_,0,*k*_ [MPa]	40.0
Mean value of density *ρ_mean_* [kg/m^3^]	550.0

**Table 3:** Mean values of Young’s modulus, the 0.2% proof strength and the tensile strength of the AW-6060 T6 aluminium alloy [37].

Parameter	Mean Value
Young’s modulus [GPa]	66.4 ± 0.51
0.2% proof strength [MPa]	181.5 ± 1.92
Tensile strength [MPa]	209.8 ± 1.05

**Table 4:** Mean values, 5%-quantiles and coefficients of variation for the 10 mm screw used in the tests.

Parameter	Mean Value	5%-Quantile	CV [%]
Shank diameter *d*_0_ [mm]	9.43	9.39	0.38
Length *L* [mm]	85.34	85.07	0.29
Outer thread diameter *d*_1_ [mm]	9.47	9.23	2.41
Inner thread diameter *d*_2_ [mm]	6.95	6.88	0.88
Pitch *p* [mm]	4.51	4.49	0.48
Thread length *L_t_* [mm]	59.64	59.04	0.96
Shank length *L_s_* [mm]	16.39	16.24	0.86
Head width across flats *F* [mm]	16.78	16.67	0.61
Head width across corners *C* [mm]	19.07	18.97	0.51
Head height *H* [mm]	6.88	6.86	0.24

**Table 5:** Mean values, 5%-quantiles and coefficients of variation for the 12 mm screw used in the tests.

Parameter	Mean Value	5%-Quantile	CV [%]
Shank diameter *d*_0_ [mm]	11.31	11.29	0.17
Length *L* [mm]	88.82	88.52	0.33
Outer thread diameter *d*_1_ [mm]	11.62	11.57	0.47
Inner thread diameter *d*_2_ [mm]	8.90	8.89	0.09
Pitch *p* [mm]	4.81	4.78	0.45
Thread length *L_t_* [mm]	62.13	61.94	0.29
Shank length *L_s_* [mm]	15.63	15.27	2.18
Head width across flats *F* [mm]	18.66	18.62	0.19
Head width across corners *C* [mm]	21.22	21.18	0.19
Head height *H* [mm]	7.89	7.84	0.57

**Table 6:** Dimensions of C2 (Bulldog) connectors ^1,2^ [41].

Connector Type	Diameter *d_c_* [mm]	Height *h_c_* [mm]	Thickness Without Zinc-Coating *t* [mm]	Hole Diameter *d*_1_ [mm]	Flange Height *h*_3_ [mm]	Number of Teeth
C2-50/M10G	50	6.6	1.00	10.4	4.0	12
C2-50/M12G	50	6.6	1.00	12.4	4.0	12

^1^ Tolerances: thickness *t* in accordance with [42,43], other dimensions: ±1.50 mm. ^2^ Limit deviations for diameter *d*_1_: plus 0.30 mm, minus 0 mm.

**Table 7:** Dimensions of C11 (Geka) connectors ^1^ (connector type: C11-50/M12) [41].

**Diameter** ***d_c_* [mm]**	**Height** ***h_c_* [mm]**	**Thickness** ***t* [mm]**	**Diameter** **of Centre Hole** ***d*_1_ [mm]**	**Diameter** **of Inner Circle** ***d*_2_ [mm]**
50	15	3	12.5	40
**Diameter** **of Spikes at Base** ***d*_4_ [mm]**	**Diameter** **of Flange** ***d*_5_ [mm]**	**Radius** ***r* [mm]**	**Height of Flange from Face** ***h*_1_ [mm]**	**Number** **of Spikes**
6	17.0	4	3	8

^1^ Tolerances on: height *h_c_*, thickness *t*, radius *r* and height of flange from face *h*_1_: ±0.50 mm, other dimensions: ±0.80 mm.

**Table 8:** The results of the push-out tests of the shear connections with 10 mm screws and without toothed-plate connectors (per one connector).

Parameter	Specimen				Mean (R10.1–R10.4)
	R10.1	R10.2	R10.3	R10.4	
*P_ult_* [kN]	17.1	16.2	17.3	16.2	16.7 ± 0.9 (5.6%)
*s_ult_* [mm]	14.4	23.0	12.4	16.9	16.7 ± 7.3 (43.9%)
*k*_0.4_ [kN/mm]	4.4	8.6	9.1	4.3	6.6 ± 4.1 (62.8%)
*k*_0.6_ [kN/mm]	4.2	7.9	7.8	4.7	6.2 ± 3.1 (51.1%)

**Table 9:** The results of the push-out tests of the shear connections with 10 mm screws and with toothed-plate connectors (type C2-50/M10G, Bulldog) (per one connector).

Parameter	Specimen				Mean (10.1–10.4)
	10.1	10.2	10.3	10.4	
*P_ult_* [kN]	20.7	20.5	22.6	22.3	21.5 ± 1.7 (8.0%)
*s_ult_* [mm]	11.7	12.3	14.0	13.0	12.8 ± 1.6 (12.3%)
*k*_0.4_ [kN/mm]	4.8	6.1	8.3	6.5	6.4 ± 2.3 (35.8%)
*k*_0.6_ [kN/mm]	5.0	6.0	6.9	5.6	5.9 ± 1.3 (21.6%)

**Table 10:** The results of the push-out tests of the shear connections with 12 mm screws and without toothed-plate connectors (per one connector).

Parameter	Specimen				Mean (R12.1–R12.4)
	R12.1	R12.2	R12.3	R12.4	
*P_ult_* [kN]	21.4	21.9	22.9	22.9	22.3 ± 1.2 (5.4%)
*s_ult_* [mm]	13.5	27.6	26.3	28.5	24.0 ± 11.2 (46.7%)
*k*_0.4_ [kN/mm]	6.8	8.9	12.4	5.9	8.5 ± 4.6 (54.1%)
*k*_0.6_ [kN/mm]	6.0	7.6	9.0	5.8	7.1 ± 2.4 (33.6%)

**Table 11:** The results of the push-out tests of the shear connections with 12 mm screws and with toothed-plate connectors (type C2-50/M12G, Bulldog) (per one connector).

Parameter	Specimen				Mean (12.1–12.4)
	12.1	12.2	12.3	12.4	
*P_ult_* [kN]	27.1	26.4	28.4	28.3	27.6 ± 1.5 (5.6%)
*s_ult_* [mm]	12.9	12.5	13.1	12.5	12.8 ± 0.5 (3.7%)
*k*_0.4_ [kN/mm]	6.2	7.7	9.2	7.0	7.5 ± 2.0 (26.9%)
*k*_0.6_ [kN/mm]	6.2	7.4	8.5	7.0	7.3 ± 1.5 (20.9%)

**Table 12:** The results of the push-out tests of the shear connections with 12 mm screws and with toothed-plate connectors (type C11-50/M12, Geka) (per one connector).

Parameter	Specimen				Mean (12.5–12.8)
	12.5	12.6	12.7	12.8	
*P_ult_* [kN]	30.8	29.7	29.7	30.0	30.1 ± 0.8 (2.8%)
*s_ult_* [mm]	8.0	7.5	8.2	7.6	7.8 ± 0.5 (6.7%)
*k*_0.4_ [kN/mm]	7.0	5.2	6.5	8.1	6.7 ± 1.9 (28.6%)
*k*_0.6_ [kN/mm]	7.2	5.8	7.0	8.0	7.0 ± 1.4 (20.7%)

## Data Availability

All data contained within the article.
